# A Ring-Augmented Roux-en-Y Gastric Bypass with MiniMizer Ring is Effective and Safe in Patients with a BMI >50 kg/m^2^

**DOI:** 10.1007/s11695-025-07988-1

**Published:** 2025-06-26

**Authors:** Marijn T.F. Jense, Kick Bluijssen, Evelien De Witte, Pieter P.H.L. Broos, Boudewijn E. Schaafsma, Jan Willem M. Greve, Evert-Jan G. Boerma

**Affiliations:** 1 Zuyderland Medisch Centrum, Sittard, Netherlands; 2 Maastricht University, Maastricht, Netherlands; 3 Maastricht University, Maastricht, Netherlands; 4 Zuyderland Medisch Centrum, Sittard, Netherlands; 5 Nederlandse Obesitas Kliniek, Zeist, Netherlands; 6 Maastricht University Medical Centre, Maastricht, Netherlands

**Keywords:** Ring-augmented gastric bypass, Banded bypass, Banded gastric bypass, Banded Roux-en-Y gastric bypass, Severe obesity, BMI over 50 kg/m^2^

## Abstract

**Background:**

The Roux-en-Y gastric bypass (RYGB) is regarded as one of the most effective treatments for obesity. The ring-augmented RYGB (raRYGB), has demonstrated superior long-term weight loss outcomes and better weight maintenance compared to the standard RYGB. There is no agreement regarding the optimal procedure for patients with a BMI >50 kg/m^2^. This study aims to evaluate the effectiveness and safety of ring augmentation of a RYGB with a MiniMizer ring in patients with a BMI >50 kg/m^2^ at 2-year follow-up.

**Methods:**

This study is a retrospective cohort study performed at the Zuyderland Medical Center in Heerlen and the Nederlandse Obesitas Kliniek Zuid. All patients with a BMI >50 kg/m^2^ who underwent a raRYGB with a MiniMizer ring in the period 2016–2021 are included. Total weight loss (TWL), obesity-related complications, and surgical-related complications were analyzed over 2 years post-operative.

**Results:**

A total of 171 patients were included, of whom 78.4% were female, with a median BMI of 54.0 kg/m^2^ (51.4–57.7 IQR) and age of 42 (32–52 IQR) years at baseline. The mean % TWL was 15.6 at 3 months, 24.8 at 6 months, 32.1 at 1 year, and 35.2 at 2 years post-operatively. Complications of Clavien Dindo 3 or higher within the first 30 post-operative days were seen in 8 patients (4.7%) of which 1 (0.6%) was ring related.

**Conclusions:**

The raRYGB with a MiniMizer ring in this study population results in high total weight loss results after 2 years. Ring-related complications rates are low. Therefore, we conclude that a raRYGB is an effective and safe treatment option for patients with a BMI >50 kg/m^2^.

## Introduction

At present, there is no consensus on the optimal surgical approach for patients with a BMI ≥ 50 kg/m^2^. This group often requires a higher percentage of total weight loss (%TWL) to achieve satisfactory weight goals and comorbidity resolution while also facing an increased risk of perioperative complications [1–4]. Various procedures have been explored for this population, including laparoscopic sleeve gastrectomy (LSG), Roux-en-Y gastric bypass (RYGB), One Anastomosis Gastric Bypass (OAGB), Single Anastomosis Duodenal Ileal Bypass (SADI), and Biliopancreatic Diversion with Duodenal Switch (BPD-DS), all with their respective pros and cons [5–7].

Augmentation of the gastric pouch of a RYGB has demonstrated superior long-term weight loss outcomes and better weight maintenance compared to standard RYGB. While numerous studies have examined various ring types, there is limited literature specifically addressing the use of silicone rings designed for raRYGB in patients with BMI ≥ 50 kg/m^2^ [8–11].

Therefore, this study aims to evaluate the effectiveness and safety of ring augmentation of RYGB with a MiniMizer ring up to 2 years after surgery in patients with BMI ≥ 50 kg/m^2^.

## Methods

In this retrospective study, all consecutive adult patients with a BMI of 50 ≥ kg/m^2^ who underwent a primary laparoscopic raRYGB with a MiniMizer ring in the Zuyderland Medical Center (ZMC) between July 2016 and December 2021 were included in this study. Prior to surgery, all patients were screened for eligibility for surgery according to IFSO criteria [12].

### Study Outcomes

The primary outcome is % TWL which is calculated using weight prior to the surgery and was investigated at intervals of 3 months, 6 months, 1 year, and 2 years after surgery. The secondary outcomes of this study comprise of post-operative complications, and obesity-related complications. All post-operative complications were scored using the Clavien Dindo (CD) classification system and were separated into early (i.e., < 30 days post-operative) or late (i.e., >30 days post-operative) complications and into regular or ring-related complications. All complications with a CD score of 3a or higher related to the surgery were extracted for analysis in the present study. Investigated obesity-related complications include diabetes mellitus, hypertension, sleep apnea syndrome (OSAS), and dyslipidemia. Improvement was determined in line with the ASMBS criteria [13].

For comparison with older literature, the percentage Excess Weight Loss (%EWL) was also calculated for every timepoint using the following formula: (baseline weight − follow up weight)/(baseline weight − Ideal Body Weight) × 100. Ideal Body Weight was set at a BMI of 25 resulting in the following formula: Weight (kg) = 25 × length (m)^2^.

### Surgical Technique

The surgical procedure of a standard laparoscopic raRYGB was performed by one of our four dedicated bariatric surgeons. The raRYGB was created with a 6–8-cm pouch, calibrated on a 40 French orogastric tube, a biliopancreatic limb of 60 cm, and an alimentary limb of 120 cm. A linear-stapler technique was used to create the gastro-jejunal and jejunal-jejunal anastomosis with suture closure of the remaining defect. Petersen’s space and the mesenteric defects created by the jejuno-jejunostomy were routinely closed with non-absorbable staples. A silicone ring, the MiniMizer Gastric Ring (Bariatric Solutions, Switzerland), was placed around the pouch, 2–3 cm below the gastro-esophageal junction and at least >2 cm above the gastro-jejunal anastomosis. The ring was fixed by two non-absorbable prolene sutures and could either be closed at 6.5, 7.0, 7.5, or 8.0 cm.

### Statistical Analysis

Data analysis was conducted using the Statistical Package for the Social Sciences (SPSS), version 26.0 (IBM Corp., Armonk, NY, USA). Descriptive statistics, including means, standard deviations, and frequencies, were calculated to summarize demographic characteristics and baseline variables of the study participants. Normality of continuous variables was assessed using the Shapiro–Wilk test. Categorical variables were analyzed using the chi-square test or Fisher’s exact test, as appropriate. The difference in obesity-related complications prevalence at different time points compared to baseline was assessed using the McNemar test. The level of statistical significance was set at *p* < 0.05. All statistical tests were two-tailed. The results are presented as mean (± standard deviation) or median (interquartile range) for continuous variables and as frequencies (percentages) for categorical variables.

## Results

### Baseline Characteristics

Between June 2016 and December 2021, a total of 171 consecutively operated patients were eligible for this study. Of these, 78% were female, with a median age of 42 years and a median BMI of 54 kg/m^2^. As outlined in Table 1, at baseline, 24 patients (14%) had diabetes mellitus, 59 patients (35%) had hypertension, 35 patients (21%) had obstructive sleep apnea syndrome (OSAS), and 14 patients (8%) had dyslipidemia.

**Table 1:** Baseline characteristics

Baseline characteristics		Total (*N* = 171)
Sex	*Female (%)*	134 (78.4)
Median age on day of operation in years (IQR)		42 (32–52)
Median BMI screening in kg/m^2^ (IQR)		54.0 (51.4–57.7)
Diabetes mellitus (%)		24 (14.0)
Hypertension (%)		59 (34.5)
OSAS (%)		36 (20.5)
Dyslipidemia (%)		14 (8.2)

*IQR* interquartile range, *BMI* Body Mass Index, *OSAS* Obstructive Sleep Apnea Syndrome

### Weight Loss

**Fig. 1:**
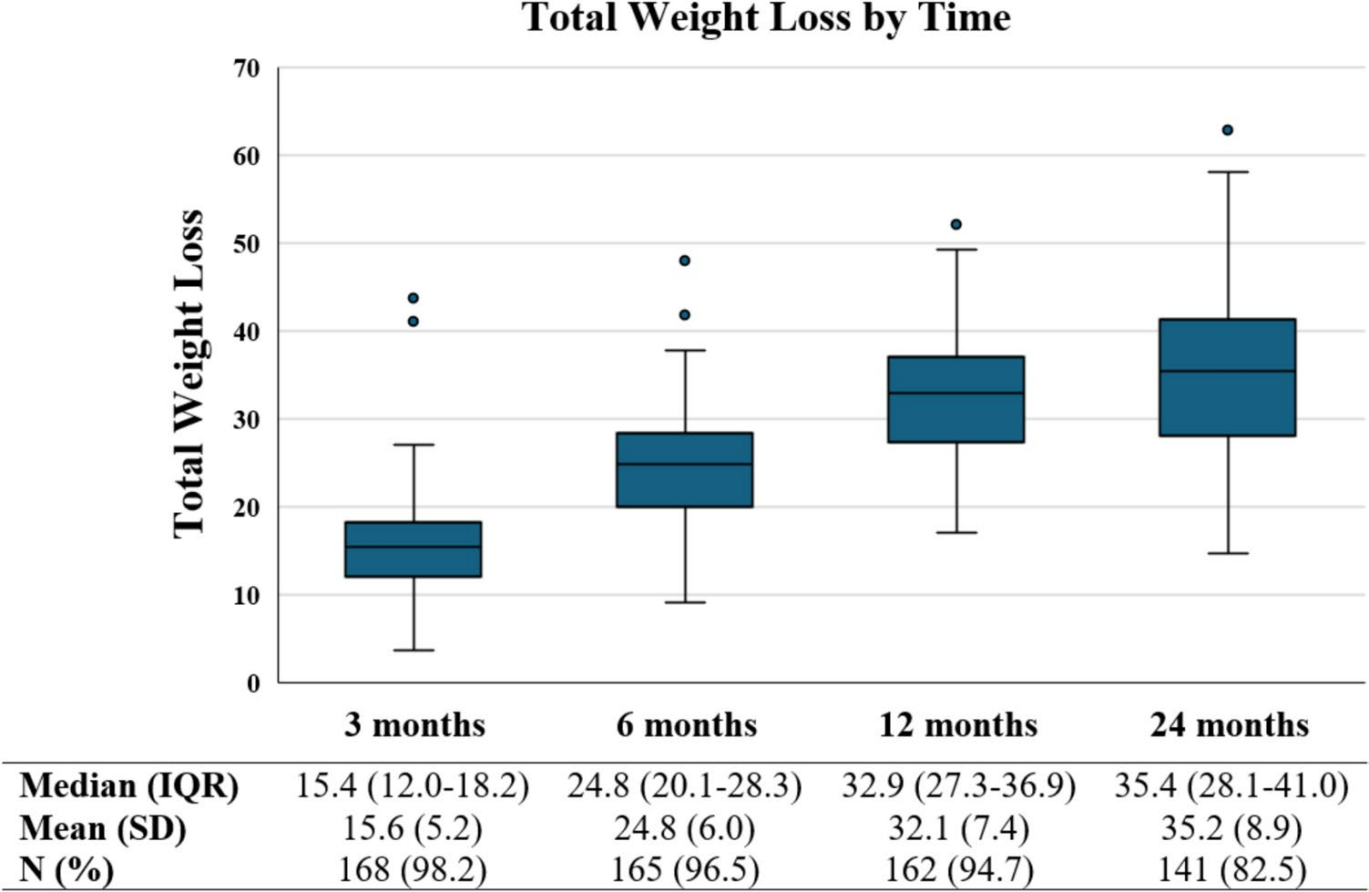
Boxplot graph presenting total weight loss results over time. IQR = interquartile range; SD = standard deviation

Mean TWL in patients with a BMI >50 kg/m^2^ who underwent a raRYGB with a MiniMizer ring was 16% (± 5.2) at 3 months, 25% (± 6.0) at 6 months, 32.1% (± 7.4) at 1 year, and 35% (± 8.9) at 2 years post-operatively, as shown in Fig. 1.

Over the course of 2 years following raRYGB surgery, there was a significant reduction in BMI, with a median BMI of 35.7 kg/m^2^ (IQR 31.8–39.0) at one year and 34.0 kg/m^2^ (IQR 30.0–38.4) at 2 years.

A table presenting delta BMI and percentage excess weight loss (%EWL) has been included to enable comparison with previously published data (Table 2).

**Table 2:** Additional weight loss measures for comparison to existing literature

		3 months	6 months	12 months	24 months
EWL %	*Mean (SD)*	29.7 (9.5)	47.6 (12.3)	61.7 (15.2)	67.7 (18.1)
	*Median (IQR)*	29.3 (22.5–35.1)	46.8 (38.3–55.1)	61.6 (51.1–72.4)	68.5 (52.2–78.8)
ΔBMI	*Mean (SD)*	10.8 (3.0)	15.5 (3.5)	19.4 (4.4)	21.2 (5.3)
	*Median (IQR)*	10.7 (8.9–12.6)	15.4 (13.2–17.6)	19.3 (16.0–22.4)	20.9 (17.2–24.1)
***N*** %		168 (98.2)	165 (96.5)	162 (94.7)	141 (82.5)

*EWL* Excess weight loss, *ΔBMI* delta BMI, *SD* standard deviation, *IQR* interquartile range

### Peri-operative Data

To optimize the success rate of the surgical procedure, patients were advised to lose weight pre-operatively using a low caloric diet for 4–6 weeks. The mean pre-operative weight loss in this study population was 8.6 (IQR 5.4–13.5) kg. The most frequent MiniMizer Gastric Ring position in male patients was 7.5 cm, observed in 91.4% of patients (*n* = 32). In the 3 other male patients the ring was closed at 7.0 cm circumference (8.6%). In 82% of the female patients, the ring position was 7.0 cm (*n* = 108). The ring was closed at 6.5 cm in 15% (*n* = 20) or 7.5 cm in 2% (*n* = 3). Only 1 female patient (1%) had the ring closed at 8.0 cm. The median operation time was 63 min (IQR 49–76), with a minimum of 23 min and a maximum of 118 min.

### Obesity-Related Complications

The percentage of patients with diabetes mellitus dropped significantly from 14% at baseline to 3.8% at 1 year and further to 3% at two years (*p* < 0.05). Hypertension prevalence decreased from 35% at baseline to 22% at 1 year and 18% at 2 years (*p* < 0.05). Similarly, the occurrence of OSAS decreased significantly from 21% at baseline to 8% at 1 year and 9% at 2 years (*p* < 0.05). Dyslipidemia prevalence showed a slight reduction, from 8% at baseline to 8% at 1 year and 5% at 2 years, although these changes were not statistically significant (Table 3).

**Table 3:** Obesity-related complications present at 1 and 2 years post-operative compared to baseline

	Obesity-related complications present				
	BMI (IQR)	Diabetes mellitus	Hypertension	OSAS	Dyslipidemia
Baseline (*n* = 171)	54.0 (51.4–57.7)	14.0%	34.5%	21.1%	8.2%
1 year (*n* = 157)	35.7 (31.8–39.0)	3.8%*	21.6%	8.3%*	7.6%
2 years (*n* = 134)	34.0 (30.0–38.4)	2.9%*	17.9%*	9.0%*	4.5%

*RYGB* Roux-en-Y gastric bypass, *BMI* Body Mass Index, *OSAS* Obstructive Sleep Apnea Syndrome

*statistical significant difference compared with baseline (tested with McNemars test. with a=0.05)

### Post-Operative Complications

Within 30 days post-operatively, 8 patients (5%) experienced complications, one (1%) of which was ring-related dysphagia. The majority of early complications consisted of anastomotic leakages (*n* = 5, 3%).

Late complications occurred in 17 patients (10%), with 2 being ring-related (1%). In one patient, a herniation of small bowel was seen through the MiniMizer, and the other patient suffered from dysphagia due to the MiniMizer. The most common late complication was internal herniation (*n* = 9, 5%). No patients underwent revisional or conversional surgery. A complete description of all complications can be found in Table 4.

**Table 4:** All Clavien Dindo 3 or higher complications within the complete study period divided by short- and long-term complications

Complications	Total (%)	< 30 days	30 days–2 years
Patients with complications	23 (13.5)	8 (4.7)	17 (9.9)
Complications	25	8	17
*Ring related*	3 (1.8)	1 (0.6)	2 (1.2)
Dysphagia*	4 (2.3)	1 (0.6)	3 (1.8)
*Anastomotic leakage*	5 (2.9)	5 (2.9)	0
*Internal herniation*	9 (5.3)	0	9 (5.3)
*Iatrogenic small bowel leak*	1 (0.6)	1 (0.6)	0
*Mechanical ileus because of adhesion*	1 (0.6)	0	1 (0.6)
*Abdominal pain*	1 (0.6)	0	1 (0.6)
*Gastro-gastric fistula*	1 (0.6)	0	1 (0.6)

*All were considered not ring-related by barium swallow

### Loss to Follow-Up

The percentage of patients lost to follow-up varied across the different time points (3 and 6 months, 1 and 2 years), ranging from 2 to 22%. All tables and figures report the amount of missing data for each measurement and time point.

## Discussion

This study presents compelling evidence for the effectiveness of ring-augmented Roux-en-Y gastric bypass (raRYGB) in patients with a BMI ≥ 50 kg/m^2^. The results demonstrate significant weight loss and improvement in obesity-related complications over a 2-year follow-up period.

### Weight Loss Outcomes

The weight loss results are particularly noteworthy, with patients achieving a mean total weight loss (TWL) of 35.2% at 2 years post-operatively. This is a substantial reduction, especially considering the high initial BMI of the patient group. The progression of weight loss from 15.6% at 3 months to 35.2% at 2 years shows a consistent and sustained weight loss trajectory. When comparing to existing literature describing RYGB, a slightly higher EWL can be found in this study’s group, with Hong et al. presenting an EWL of 66.4% after 2 years and Junior et al. presenting an EWL of 66% [14, 15]. Besides, when one compares the weight loss results of this study to existing literature, one can see that the TWL results in the raRYGB are higher when compared to RYGB, with a TWL of 31.1% in the study by Mustafa et al., although the study by Enochs et al. presents a TWL of 35.2% for patients with a BMI of 45–55 kg/m^2^ or 39% for patients with a BMI of 55 kg/m^2^ [16, 17]. When comparing to SADI-s, one can see a slightly higher TWL of 37.8%. However, the study by Surve et al. also presents higher complications rates of 22.9% [18].

Since this is a single-arm study, no direct comparison can be made. However, there is an article published on data from this particular center. This article shows a TWL of 35% after 2 years for the raRYGB. In this article, a comparison to RYGB is made, which shows a TWL of 33% [19].

### Obesity-Related Complication Improvement

The present study reports significant improvements in obesity-related complications. These reductions in obesity-related complications are statistically and clinically significant. When comparing this study’s results to existing literature such as the review of Balamurugan et al., one can see that the outcome is comparable to the results of RYGB, AOGB, and SADI-s. This review presents a remission rate of diabetes of 73.8%, 76.9, and 89.5% in RYGB, AOGB, and SADI-s. The present study shows a remission of 83.3% for diabetes. For hypertension, the present study shows 59.3%, compared to 60.6% and 64.3% in RYGB and AOGB, respectively. Dyslipidemia remission was seen in 57% of patients in the present study compared to 82.4% and 71.8% in RYGB and OAGB [20].

### Complications

The overall complication rate was relatively low, with 4.7% early complications and 9.4% late complications, which was comparable to the complications rate described in the article of Mognol et al. [21]. This is encouraging, considering the higher risk profile of patients with BMI ≥ 50 kg/m^2^. The most common late complication was internal herniation (5.3%), which is a known risk in RYGB procedures, possibly because closing the mesenteric and mesocolic defect created with the RYGB is more difficult in patients with a higher BMI. Furthermore, patients can lose more weight, which can lead to the opening of the defects.

The low rate of ring-related complications, only 3 cases, suggests that this technique is relatively safe when performed by experienced surgeons.

### Surgical Considerations

Currently, the sleeve gastrectomy is one of the most preferred bariatric procedures in patients with a BMI of 50 or higher [1]. The sleeve gastrectomy is easier to perform in these patients since less abdominal space is necessary. Furthermore, the sleeve gastrectomy can be performed faster than the RYGB, resulting in shorter operating time and reducing the risks that can come with anesthesia [22]. While these arguments are valid, the sleeve procedure also has drawbacks, including a higher likelihood of recurrent weight gain over time and less favorable weight loss outcomes compared to RYGB, which may ultimately necessitate revisional or conversion surgery [23, 24]. There are also concerns regarding two-step bariatric procedures, such as staged SADI, as it inherently requires a planned second operation.

Augmentation of the RYGB with the MiniMizer ring results in higher weight loss results and better weight maintenance over time, hypothetically leading to less revisional or conversional operations and with that potentially reduces the risks of complications over time [25, 26].

### Limitations and Future Directions

While the results are promising, the study has some limitations. The follow-up period of 2 years, while informative, does not capture long-term outcomes. The loss to follow-up, reaching 21.6% at some time points, could potentially bias the results. The study lacks a control group, making it difficult to directly compare raRYGB to other procedures in this high-BMI population.

Future research should focus on longer-term follow-up and comparative studies with other bariatric procedures for patients with BMI ≥ 50 kg/m^2^. Additionally, investigating factors that contribute to the success of raRYGB in this population could help refine patient selection and surgical techniques.

## Conclusion

In conclusion, this study provides valuable evidence supporting the use of raRYGB with a MiniMizer ring in patients with BMI ≥ 50 kg/m^2^, demonstrating significant weight loss and improvement in obesity-related complications with an acceptable complication rate.

## Data Availability

No datasets were generated or analysed during the current study.
